# Surgical Management of Destructive Thyroiditis Triggered by Neoadjuvant Immune Checkpoint Inhibitor Therapy in Locally Advanced Non-Small Cell Lung Cancer: A Case Report

**DOI:** 10.70352/scrj.cr.25-0104

**Published:** 2025-05-28

**Authors:** Sachi Kawagishi, Toru Kimura, Kenji Kimura, Eriko Fukui, Takashi Kanou, Naoko Ose, Yasushi Shintani

**Affiliations:** Department of General Thoracic Surgery, The University of Osaka Graduate School of Medicine, Suita, Osaka, Japan

**Keywords:** non-small cell lung cancer, destructive thyroiditis, immune-related adverse event, surgery

## Abstract

**INTRODUCTION:**

The use of immune checkpoint inhibitors (ICIs) as neoadjuvant therapies for locally advanced and resectable non-small cell lung cancer is increasing. As a result, immune-related adverse events (irAEs) may be observed before surgery and may require preoperative intervention. We report the case of a patient with destructive thyroiditis induced by neoadjuvant ICI treatment, in which surgical resection was performed after steroid treatment.

**CASE PRESENTATION:**

A 74-year-old woman was diagnosed with slow-growing squamous cell carcinoma of the right upper lobe during treatment for another disease. Imaging studies revealed a small nodule suggestive of pulmonary metastasis in the right upper lobe and hilar lymph node metastasis. The patient was initially diagnosed with primary lung cancer of the right upper lobe (cT3N1M0, Stage IIIA, TNM Classification, 8th edition), and neoadjuvant nivolumab combined with chemotherapy was planned every 3 weeks for three cycles. After the first cycle, the patient experienced drug-induced kidney injury. Nivolumab and chemotherapy were discontinued, and surgical resection was planned. However, a laboratory analysis on the day before surgery revealed elevated free triiodothyronine and free thyroxine, and decreased thyroid-stimulating hormone. Subsequent examination led to a diagnosis of destructive thyroiditis due to irAEs, and surgery was postponed. Dexamethasone was administered orally for 1 week, and once the thyroid function showed consistent improvement, a thoracoscopic right upper lobectomy was performed. The patient progressed without any other complications after surgery.

**CONCLUSIONS:**

This report highlights a case of preoperative destructive thyroiditis secondary to irAEs. In patients receiving preoperative ICIs therapy, routine blood tests, including thyroid function tests, are recommended as part of preoperative assessment. In this case, the patient underwent lobectomy safely following steroid administration. The optimal timing of surgery in patients with preoperative ICI-induced destructive thyroiditis requires further investigation.

## Abbreviations

FDG-PET 18F
: fluorodeoxyglucose positron emission tomography
FT3
: free triiodothyronine
FT4
: free thyroxine
irAEs
: immune-related adverse events
ICIs
: immune checkpoint inhibitors
NSCLC
: non-small cell lung cancer
TPOAb
: thyroperoxidase antibodies
TgAb
: anti-thyroglobulin antibody
TSH
: thyroid-stimulating hormone

## INTRODUCTION

ICIs are an effective treatment option for advanced malignancies.^1,2)^ Neoadjuvant nivolumab combined with chemotherapy has been shown to improve outcomes in patients with resectable and locally NSCLC.^3)^ In cases of preoperative irAEs, the timing of surgical resection remains unclear. We herein report the case of a patient who was able to safely undergo surgery for right upper lobectomy after the administration of steroids for ICI-induced destructive thyroiditis.

## CASE PRESENTATION

**Fig. 1:**
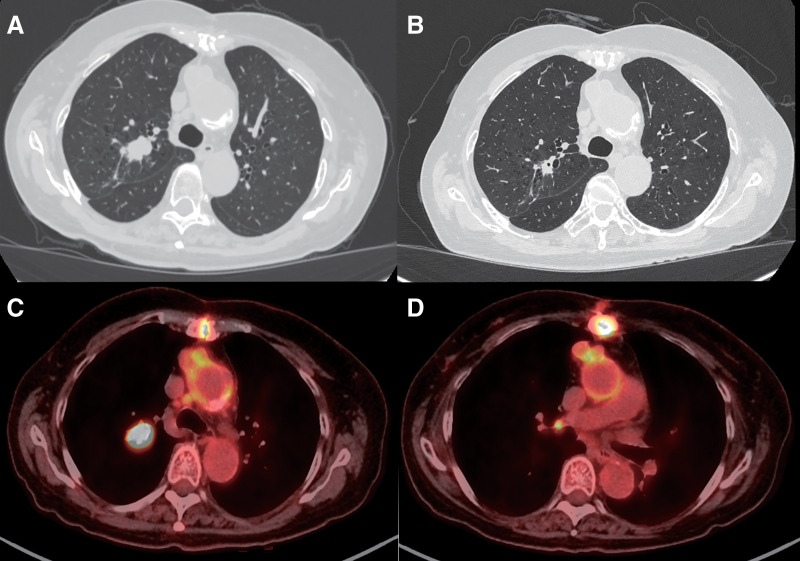
(**A**, **B**) Chest CT images obtained before (**A**) and after (**B**) neoadjuvant chemotherapy. A tumor in the right upper lobe decreased in size from 23 mm to 8 mm. (**C**, **D**) ^18^F-fluorodexyglycose positron emission tomography/computed tomography (^18^F-FDG PET/CT) findings before treatment. Increased FDG uptake is observed in the right upper lobe tumor (**C**; SUVmax = 29.43) and in the right hilar lymph node (**D**).

During a close examination for impaired consciousness, a 74-year-old woman was diagnosed with type A acute aortic dissection. Preoperative chest CT revealed an incidental nodule in the right upper lobe. At the 3-month follow-up after surgery for aortic dissection, chest CT showed that the nodule was 23 mm in size, with another 5-mm nodule in the right upper lobe, indicative of intrapulmonary metastasis (**Fig. 1A**). FDG-PET/CT showed uptake in the right upper lobe nodules (SUVmax = 29.43) and right hilar lymph node (**Fig. 1C**, **1D**). Bronchoscopy of the pulmonary nodules revealed squamous cell carcinoma (PD-L1 TPS, 99%). The clinical stage was diagnosed as T3N1M0 stage IIIA (TNM Classification, 8th edition). Neoadjuvant chemotherapy and surgery were then performed. The patient had a history of oral treatment for Graves’ disease 20 years prior but was not receiving treatment at the time of her current presentation. Testing of the thyroid function before the start of nivolumab treatment showed her levels were within the normal ranges (FT3, 1.4 pg/mL [normal range: 2.1–3.1 pg/mL]; TSH, 1.36 μIU/mL [normal range: 0.61–4.23 μIU/mL]). Four cycles of nivolumab (360 mg), carboplatin (350 mg), and paclitaxel (250 mg) were administered as neoadjuvant chemotherapy. After the first cycle, she developed drug-induced kidney injury (creatinine: 0.84 mg/dL before neoadjuvant chemotherapy, 1.28 mg/dL after the first cycle). Chest CT revealed that the right upper lobe nodule had been reduced to 8 mm in size (**Fig. 1B**). After a discussion with the multidisciplinary lung team, including our department, chemotherapy was discontinued owing to drug-induced nephropathy, and surgery was planned.

**Fig. 2:**
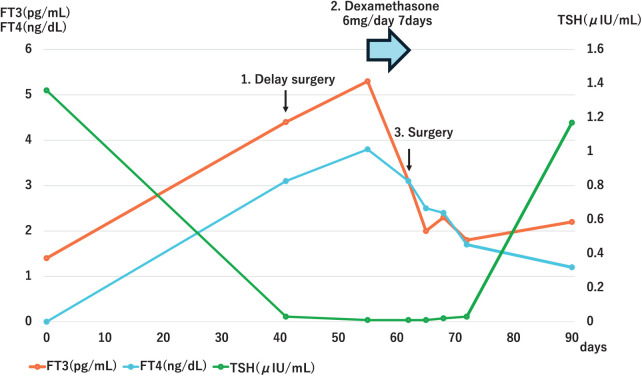
Time course of the thyroid function of the patient during treatment. Day 0 represents baseline thyroid function tests prior to the first cycle of neoadjuvant chemotherapy. (1) Preoperative blood tests performed one day before surgery revealed hyperthyroidism. The patient was diagnosed with ICI-induced destructive thyroiditis, and the surgery was postponed. (2) Two weeks later, thyroid function had further deteriorated, and oral dexamethasone was initiated. (3) One week after starting treatment, blood tests demonstrated a trend toward improvement in the patient’s FT3 and FT4 levels. Following this, the patient underwent surgery. FT3, free triiodothyronine; FT4, free thyroxine; ICI, immune checkpoint inhibitor; TSH, thyroid-stimulating hormone

The preoperative blood, performed 6 weeks after nivolumab treatment and 1 day before surgery, indicated hyperthyroidism (TSH, 0.03 μIU/mL; FT3, 4.4 pg/mL; FT4, 3.1 ng/dL [normal range 0.8-1.7 ng/dL]) (**Fig. 2**(1)). The patient showed no significant subjective symptoms and her vital signs were stable. The patient was referred to the Department of Metabolic Medicine for examination. Additional blood tests revealed that her TPOAb and TgAb titers were within the normal range (3.7 IU/mL [normal range, <5.6 IU/mL] and <5.0 IU/mL [normal range, <5.0 IU/mL]), respectively. An ultrasound examination of the thyroid gland revealed enlargement but no hypervascularization. Based on these results, the patient was diagnosed with ICI-induced destructive thyroiditis and not an exacerbation of Graves’ disease. Surgery was postponed because of the risk of a perioperative thyroid crisis. At an outpatient visit 2 weeks later (8 weeks after the administration of adjuvant chemotherapy), the thyroid function had further deteriorated (**Fig. 2**(2)). With regard to tumor regrowth, prompt management of the thyroid disorder was prioritized to enable surgical resection as soon as possible. After consulting with the Department of Metabolic Medicine, the patient was prescribed oral dexamethasone (6 mg, once daily, for 1 week) to ensure no deterioration of the thyroid function before scheduling surgery.

After 1 week, blood tests showed a trend toward improvement in the patient’s FT3 and FT4 levels (**Fig. 2**(3)). Subsequently, the patient underwent a thoracoscopic right upper lobectomy and lymph node dissection. The postoperative course was uneventful without deterioration of her thyroid function, and the patient was discharged 13 days after surgery. The postoperative pathological findings revealed no malignant cells in the primary tumor or adjacent lung tissue; however, an examination of the hilar lymph nodes revealed squamous cell carcinoma (PD-L1 TPS: 70%). The TNM classification was yp-T0N1M0 stage IIB. Since the patient’s discharge, her thyroid function normalized (**Fig. 2**), and the patient experienced no recurrence for 7 months after neoadjuvant chemotherapy and 5 months after surgery.

## DISCUSSION

ICIs are an effective treatment option for various advanced malignancies, including NSCLC.^1,2)^ As neoadjuvant nivolumab combined with chemotherapy has been shown to improve outcomes in patients with resectable and locally advanced NSCLC, the use of neoadjuvant ICIs is becoming increasingly common.^3)^ However, it is important to note that treatment with ICIs can lead to irAEs because of excessive immune activation in normal tissues. IrAEs can affect a wide range of organs, with thyroid disorder and hypophysitis as the most common endocrine-related irAEs. Thyroid disorders induced by ICIs include thyrotoxicosis (manifesting as autoimmune hyperthyroidism and/or destructive thyroiditis) and hypothyroidism.^4–6)^ Destructive thyroiditis is a condition in which thyroid tissue is destroyed for some reason, and thyroid hormone is released into the blood, leading to increased levels of thyroid hormone and suppressed TSH.^7)^ Blood tests typically show elevated levels of FT4 and FT3, with suppressed or undetectable TSH. TgAb and TPOAb are negative in most cases. Color Doppler ultrasonography is useful for differentiating destructive thyroiditis, which demonstrates reduced or absent intrathyroidal blood flow, from conditions associated with thyroid hyperfunction, typically show increased vascularity.^8)^ In the present case, these characteristic findings were observed on both blood tests and ultrasonography. Additionally, absent radionuclide uptake on 99 m-technetium scintigraphy is considered a hallmark of destructive thyroiditis.^9)^ A previous report indicated that the majority of cases of thyrotoxicosis, as irAEs, are transient and result from destructive thyroiditis, which is typically followed by persistent hypothyroidism.^4,5)^ Another study found that a history of thyroid-related disease or elevated TSH levels prior to treatment is associated with an increased risk of developing thyroid dysfunction as an irAE.^10)^ The development of thyroid irAEs is associated with prolonged survival in patients with NSCLC and may be predicted by baseline antithyroid antibodies.^6)^ Destructive thyroiditis and hypothyroidism, irAEs, are unlikely to cause noticeable symptoms and are often identified through abnormal blood test results.^11)^

When ICIs are administered as part of neoadjuvant chemotherapy and thyroid dysfunction is observed, the decision to proceed with surgery can significantly impact the outcomes of treatment for NSCLC. Forde et al. reported that hypothyroidism or thyroiditis occurred in 2.3% of patients with Stage IBIIIA resectable NSCLC who received nivolumab; however, details of subsequent treatment courses were not provided.^3)^ Another report found that some patients discontinued ICIs because of thyroid dysfunction as an irAE and were unable to undergo resection surgery because of tumor progression.^12)^ To the best of our knowledge, there are currently no reports of surgery in patients with destructive thyroiditis after neoadjuvant ICIs treatment for resectable NSCLC.^13)^ Thyroid dysfunction induced by ICIs is rarely reported to progress to a thyroid crisis, while untreated or inadequate treatment of hyperthyroidism can generally trigger a thyroid crisis, and nonthyroidal surgery is one of the risk factors that can induce a thyroid crisis.^14,15)^ Thyroid crisis is a life-threatening condition resulting from excessive thyroid hormone levels. A previous report described an ICI-induced case in advanced NSCLC that required norepinephrine, invasive ventilation for circulatory failure, and continuous hemodiafiltration for acute renal failure.^16)^ The clinical management of hyperthyroidism as an irAE in the preoperative setting remains poorly understood.

Autoimmune hyperthyroidism is persistent and requires anti-thyroid treatment, which is ineffective for destructive thyroiditis.^17)^ Destructive thyroiditis is usually transient and improves spontaneously, with the subjective symptom of tachycardia being manageable with beta-blocker therapy.^4)^ The time required for spontaneous improvement of destructive thyroiditis ranges from a few days to a few weeks and varies depending on the case.^4,5)^ Ma et al. reported that the median duration of thyrotoxicosis in patients with destructive thyroid was 42 (range: 14273) days.^5)^ While current guidelines recommend no treatment because of its self-limiting nature, a short course of oral prednisone (25 mg/day for 3 weeks) has shown promise in the management of severe cases, particularly in patients with enlarged thyroid volumes and a poor performance status.^18)^ Because of variations in corticosteroid dosages reported in the literature, the optimal dosage for the treatment of destructive thyroiditis has not yet been clearly established.^5,18)^ By limiting the inflammatory destruction of thyroid tissue caused by ICIs and reducing the release of thyroid hormones (FT4 and FT3) into the bloodstream, this steroid treatment significantly lowers thyroid hormone levels and shortens the time to remission without affecting tumor progression or hypothyroidism rates at 6 months.^18)^ Additionally, glucocorticoids suppress type I deiodinase (D1) activity, reducing the peripheral conversion of T4 to active T3, while stimulating type III deiodinase (D3) activity, which increases the conversion of T4 to reverse T3 (inactive form), thereby lowering active thyroid hormone levels and mitigating symptoms of thyrotoxicosis.^19)^

In our case, a preoperative examination revealed destructive thyroiditis following the administration of nivolumab combined with chemotherapy, which was discontinued after the first cycle due to drug-induced nephropathy. Our case involved locally advanced NSCLC, and tumor regrowth during prolonged preoperative period was a concern. Indeed, postoperative pathological examination revealed viable cancer cells in the hilar lymph nodes, suggesting a potential risk of tumor progression prior to surgical resection. As the thyroid function further deteriorated rather than improved during the 2-week follow-up period, we administered a brief course of steroid treatment to achieve rapid remission of thyrotoxicosis, enabling us to successfully resect the tumor. Postoperatively, the patient progressed without complications, including a thyroid crisis. Further studies are needed to establish the optimal timing of surgery in similar cases. Many patients with destructive thyroiditis as an irAE develop hypothyroidism and require thyroid hormone replacement therapy.^5)^ Consistent follow-up examinations of the thyroid function are essential, as was demonstrated in this case.

## CONCLUSIONS

This report highlights the case of a patient who safely underwent surgery after steroid therapy for ICI-induced destructive thyroiditis. As destructive thyroiditis is typically asymptomatic but can lead to thyroid crisis, preoperative blood testing, including thyroid function, is essential following neoadjuvant ICI treatment. This case underscores the importance of early recognition and appropriate preoperative management of thyroid dysfunction. As demonstrated in this case, the optimal timing of surgery in patients with preoperative destructive thyroiditis caused by ICIs requires further investigation.

## DECLARATIONS

### Funding

This study was not funded.

### Authors’ contributions

SK conceptualized and designed the article.

All the authors have read and approved the final version of the manuscript.

### Availability of data and materials

The patient data in this case report will not be shared to ensure patient confidentiality. The derived data supporting the findings of this study are available from the corresponding author on reasonable request.

### Ethics approval and consent to participate

This study was approved by the Ethical Review Board of Osaka University Hospital (Approval No. 18528). Written informed consent for participation in this case report was obtained from the patient.

### Consent for publication

Informed consent for publication of this case report was obtained from the patient.

### Competing interests

The authors declare no conflicts of interest in association with the present study.
